# Variable expression of hepatic genes in different liver tumor cell lines: conclusions for drug testing

**DOI:** 10.3389/fcell.2025.1646602

**Published:** 2025-09-02

**Authors:** Monika Joanna Wisniewska, Agnieszka Wencel, Malgorzata Jakubowska, Joanna Agata Motyl, Krzysztof Dudek, Beata Burzynska, Dorota Genowefa Pijanowska, Krzysztof Dariusz Pluta

**Affiliations:** ^1^ Department of Hybrid and Analytical Microbiosystems, Nalecz Institute of Biocybernetics and Biomedical Engineering, Polish Academy of Sciences, Warsaw, Poland; ^2^ Department of General, Transplant & Liver Surgery, University Medical Center, Medical University of Warsaw, Warsaw, Poland; ^3^ Institute of Biochemistry and Biophysics, Polish Academy of Sciences, Warsaw, Poland

**Keywords:** liver tumor cell lines, hepatic gene expression, hepato-and cytotoxicity testing, hepatocellular carcinoma, hepatoblastoma

## Abstract

**Introduction:**

Drug discovery and development is a complex, multi-stage process that often spans over a decade and involves high costs and low success rates. Preclinical testing, particularly the assessment of drug-induced liver injury, plays a crucial role in identifying safe and effective therapeutics before clinical trials. *In vitro* models based on hepatic cell lines are commonly used to study hepatotoxicity, yet their physiological relevance varies significantly. This study aimed to compare the expression of key liver-specific genes and proteins in four widely used hepatic cancer cell lines—HepG2, C3A, SNU449, and SNU475—with those in primary human hepatocytes.

**Methods:**

Using RT-qPCR, protein analysis and metabolic tests, we assessed the ability of these cell lines to perform liver-specific functions, especially drug metabolism.

**Results:**

We found significant differences in liver-related gene expression and metabolic profiles among tested cell lines, especially the most striking differences were found between tumor cells of divergent origin: hepatoblastomas and hepatocellular carcinomas.

**Discussion:**

Our findings emphasize the importance of careful selection and validation of *in vitro* models in hepatotoxicity testing, as significant differences exist in their gene expression profiles and functional characteristics.

## 1 Introduction

Successful drug discovery and development is a very challenging and complex process that consists of five stages. The first stage, called the prediscovery stage, covers basic research, which aims to understand the mechanisms of different diseases and finally identify potential target molecules. During the second step, named the drug discovery stage, therapeutic agents, which affect the investigated disease by interacting with target proteins, have been developed. The third stage consists of preclinical research on the most promising therapeutic agents via *in vitro* and *in vivo* models. The fourth stage includes three phases of clinical trials, which are performed on humans to evaluate the efficacy and, most importantly, the safety of the therapeutic drug. The fifth stage involves reviewing the results of clinical research by the Food and Drug Administration (FDA) or other regulatory organs and finally making decisions about the approval of the new medicament on the market. This last step also includes postmarket monitoring of potential side effects and adverse events of the released drug (Mohs and Greig, 2017; Kandi and Vadakedath, 2023; Singh et al., 2023). The whole procedure is very expensive and time-consuming because it lasts, on average, 10–15 years (Sun et al., 2022). Moreover, it is worth noting that the success rate of the introduction of the new medicament on the market is relatively low. According to the statistical resources, currently, the total number of new drugs in the R&D pipeline worldwide is 22,825. However, approximately 50 new substances are released annually into the market. For example, in 2023, the FDA approved only 55 new medicaments on the market (Statista, 2025; de la Torre and Albericio, 2024; Shareef et al., 2024).

Before potential therapeutic drugs are tested in human volunteers, preclinical research is absolutely indispensable. In general, during this study, the efficacy and safety of the analyzed therapeutic medicaments were investigated. More precisely, in *in vitro* and *in vivo* models, research on the toxicology, pharmacodynamics, and pharmacokinetics of drug candidates has been performed (Honek, 2017). Notably, studies on drug toxicity, especially drug hepatotoxicity, are extremely important. The metabolism of drugs and xenobiotics occurs in the liver. When the tested drug (or its metabolites) causes hepatotoxicity, abnormal biotransformation of drugs and other adverse reactions occur, which leads to hepatic dysfunction called drug-induced liver injury (DILI) and, in many cases, acute liver failure (ALF). Therefore, the ability to predict drug hepatotoxicity in the early stage of development is highly important (Fisher et al., 2015; Segovia-Zafra et al., 2021). Thus, another vital aspect is the choice of appropriate *in vitro* and *in vivo* models to study hepatotoxicity. Currently, human and animal cell-based *in vitro* and animal *in vivo* models are used in drug toxicity research. However, the literature reports poor predictability of animal models due to many discrepancies between human and animal species, for example, in proteins engaged in drug metabolism (Heinonen, 2015).

To study drug hepatotoxicity, *in vitro* cell-based models should meet certain conditions. First, they should be characterized by membrane polarity because such a configuration enables the proper functionality of drug transporters to be maintained. Moreover, they should demonstrate the activity of phase I and phase II drug-metabolizing enzymes, which are responsible for xenobiotic biotransformation (Han et al., 2019). On the basis of the abovementioned features, the most suitable cells for studying drug hepatoxicity are human liver parenchymal cells - hepatocytes. However, the following obstacles, such as poor availability, lack of the capacity to proliferate, and quick dedifferentiation *ex vivo* with the loss of the ability to perform liver-specific functions, limit their usage in preclinical studies (Rowe et al., 2013). Therefore, many attempts to preserve the functionality of human hepatocytes *in vitro* have been undertaken, including cocultures with nonparenchymal cells, 3D cultures, additives to the culture medium (e.g., growth factors) or the utilization of different growth substrata, which enhance cell adhesion. Nevertheless, as the availability of human hepatocytes is limited, scientists have focused on alternative sources of cells, such as cell lines derived from different primary liver cancers (hepatomas): hepatoblastomas and hepatocellular carcinomas (HCCs), or hepatocyte-like cells derived from stem cells, which could constitute a model of human liver parenchymal cells (Gómez-Lechón et al., 2014; Ruoß et al., 2020; Pluta et al., 2021).

Currently, cancerous liver-derived cell lines are widely used in drug hepatotoxicity research. The most commonly used cell line is HepG2; however, the number of available cell lines is enormous. The other widely used cell lines are HepaRG, Huh7 or Hep3B (Donato et al., 2013). Notably, the functionality of different human tumor liver cell lines is not comparable to that of liver parenchymal cells. For example, the HepG2 cell line derived from human hepatoblastoma (thus far mistakenly thought to originate from human HCC) was isolated in 1975 and is characterized by a lack (e.g., CYP1A2, CYP2C9) or low (e.g., CYP3A4, UGT1A1) expression of phase I- and phase II-metabolizing enzymes. Only the phase II enzymes SULT1A1 and SULT2A1 are expressed at levels similar to those of human hepatocytes (Aden et al., 1979; López-Terrada et al., 2009; Wiśniewski et al., 2016). Moreover, HepG2 cells and its clonal derivative - C3A, similar to HCC cell lines (e.g., SNU449, SNU475, Hep3B, and Huh7), have an unfunctional urea cycle, which results from low or undetectable expression of genes and proteins involved in this metabolic process. Interestingly, scientific data revealed that this is a common feature of HCC tumors. Therefore, it is suggested that HepG2 cells should be used for testing anticancer drugs instead of evaluating the potential hepatotoxicity of newly developed drugs (Pluta et al., 2020; Missiaen et al., 2022; Jakubowska et al., 2024). Another cancer cell line, Hep3B, is derived from primary HCC and is widely known as an *in vitro* model for investigating the mechanism of hepatotoxicity caused by acetaminophen (APAP). In contrast to HepG2 cells, Hep3B cells are positive for hepatitis B virus (HBV) and have tumorigenic potential. Moreover, they are characterized by the presence of fewer proteins specific for human hepatocytes and different responses to analyzed drugs than HepG2 cells. A possible explanation for this phenomenon is the different origins of the 2 cell lines (derived from hepatoblastoma and HCC, respectively), which results in different phenotypes: Hep3B cells present more features characteristic of mesenchymal cells (fibroblasts), whereas HepG2 cells present more features characteristic of hepatocytes (Manov et al., 2002; Slany et al., 2010; Qiu et al., 2015). In turn, the HuH7 cell line is widely used in research concerning hepatitis C virus (HCV) infection because of its high susceptibility to this pathogen. Moreover, this HCC cell line has also been utilized in drug cytotoxicity studies; however, reports in the literature have shown that the mRNA levels of phase I- and phase II-metabolizing enzymes are extremely low compared with those in human hepatocytes (with the exception of the CYP1A1 gene) (Nakabayashi et al., 1982; Choi et al., 2009). Nevertheless, the most promising cell line for studying potential drug hepatotoxicity is HepaRG cells. These cells are derived from HCC and are able to exhibit two different cell phenotypes—hepatocyte-like or biliary-like. Importantly, the majority of HepaRG cells described in the literature express phase I- and phase II-metabolizing enzymes (e.g., CYP3A4, CYP1A2, CYP2C9, and CYP2E1) at levels more comparable to those in human hepatocytes than those in the HepG2 cell line (Gripon et al., 2002; Guillouzo et al., 2007).

As previously mentioned, different hepatic cell lines utilized in drug hepatoxicity assays may significantly differ from human liver parenchymal cells and from each other. Therefore, the aim of our research was to demonstrate that before a hepatic cell line is selected for preclinical studies, it is necessary to investigate and compare the expression of genes and proteins, which are crucial in assessing the hepatotoxicity of the analyzed drug. In this report, we compared the expression of selected relevant liver-related genes in HepG2, C3A, SNU449, and SNU475 cell lines using RT-qPCR analysis. Additionally, we analyzed the differences in their phenotype and the ability to perform liver-specific functions. Finally, we investigated the expression of liver-related proteins, especially those engaged in drug metabolism, and compared with results obtained for isolated human hepatocytes.

## 2 Materials and methods

### 2.1 Cell cultures

The human liver cancer cell lines: HepG2 (HB-8065), C3A (CRL-10741), SNU449 (CRL-2234), and SNU475 (CRL-2236) were obtained from the American Type Culture Collection (ATCC, Manassas, VA, United States). Cell cultures were performed using the following media: high glucose Dulbecco’s Modified Eagle Medium (DMEM) (Sigma Aldrich, Poznan, Poland) supplemented with 1% nonessential amino acids solution (Biological Industries Inc., Beit-Haemek, Israel); Eagle’s Minimum Essential Medium (EMEM) (30–2003, ATCC, Manassas, VA, United States); and RPMI-1640 (30–2001, ATCC, Manassas, VA, United States). All the culture media used were supplemented with 10% fetal bovine serum (FBS) (30–2020, ATCC, Manassas, VA, United States).

Human Plateable Hepatocytes (HH) (Cat. No. HMCPTS) were obtained from Gibco (Thermo Fisher Scientific, Waltham, MA, United States) and cultured in William’s E Medium, GlutaMAX Supplement (Thermo Fisher Scientific, Waltham, MA, United States) supplemented with 4 μg/mL insulin from bovine pancreas and 50 μM hydrocortisone (both from Sigma Aldrich, Poznan, Poland).

Liver cell isolates (LCI) were obtained from resected fragments of human livers as described elsewhere (Zakrzewska et al., 2017). Biological material was obtained in cooperation with the Department of General, Transplant and Liver Surgery, University Medical Center, Medical University of Warsaw, Poland. Ethical approval for the study was granted by the local research ethics committee (reference number KB/182/2008). The isolated cells were cultured using a commercially available kit (ScienCell, Cat. No. 5201, Carlsbad, CA, United States), which consists of basal medium - Hepatocyte Medium (HM) supplemented with hepatocyte growth supplement (HGS, Cat. No. 5252), 10% FBS, Cat. No. 0025), and antibiotic solution (P/S, Cat. No. 0503).

Due to the limited number of HH and LCI cultures repetitions (n = 1), results for these cell types were used only illustratively and were not subject to statistical analysis.

All the cell cultures were cultured under standard conditions (in an atmosphere containing 5% CO_2_ at 37 °C).

### 2.2 Cell viability and doubling time

To assess cell viability, cells at a density of 1.5 × 10^5^ per well were seeded on a 12-well plate and cultured for 4 days without changing the medium. The cells were subsequently detached with 0.25% trypsin-EDTA (Biological Industries, Beit-Haemek, Izrael) and counted in a Burker chamber via the trypan blue exclusion method.

### 2.3 2-(4,5-Dimethylthiazol-2-yl)-2,5-diphenyltetrazolium bromide (MTT) assay

To test cellular metabolic activity, 3 × 10^4^ cells per well were seeded into a 96-well plate and cultured for 4 days without changing the medium. After that, MTT (Sigma Aldrich, Poznan, Poland) was added at a final concentration of 0.25 mg/mL per well, and the mixture was incubated for 2 h at 37 °C. Then, 150 µL of isopropanol (CHEMPUR, Piekary Slaskie, Poland) was added to each well and incubated for 15 min at 37 °C. The absorbance was measured at a wavelength of 570 nm via a Synergy HT Bio-Tek microplate reader (BIOKOM, Janki, Poland).

### 2.4 Apical vacuoles

To test the capacity of the cells to form apical vacuoles, the cells were seeded at a density of 8 × 10^4^ per well on a 24-well plate and cultured for 3 days. The cells were subsequently fixed with 4% formaldehyde (Sigma Aldrich, Poznan, Poland) and permeabilized with 0.1% Triton-X (Sigma Aldrich, Poznan, Poland). Next, the samples were treated with an F-actin Staining Kit–Red Fluorescence–Cytopainter (Abcam, Cambridge, United Kingdom), and the cell nuclei were stained with DAPI (Sigma Aldrich, Poznan, Poland). Microphotographs were taken using Olympus IX71 fluorescence microscope (Olympus, Warsaw, Poland) at a magnification of 20x. Insert microphotograph was taken using confocal laser scanning microscope FLUOVIEW^TM^ FV4000 (Evident Europe GmbH) at a magnification of 60x.

### 2.5 ELISA

A sandwich enzyme-linked immunosorbent assay (ELISA) with a quantification kit (Bethyl Laboratories, INC., Montgomery, TX, United States) was used to determine the concentration of human albumin secreted by the tested cells into the culture media. The cells were seeded at a density of 1 × 10^6^ in a T25 culture flask and cultured for 7 days. After 2, 4, and 7 days of culture, the medium samples were collected, and fresh medium was added. A Synergy HT Bio-Tek microplate reader (BIOKOM, Janki, Poland) was used to read the absorbance of the samples at a wavelength of 450 nm.

### 2.6 Western blot analysis

To prepare protein samples, cells were seeded at a density of 3 × 10^6^ on a 6 cm culture dish and cultured for 2 days. Then, the cells were collected by scratching and suspended in RIPA buffer (Thermo Fisher Scientific, Waltham, MA, United States) supplemented with inhibitors of proteases, phosphatases, and EDTA (Halt Protease Inhibitor Cocktail 100x; Halt Phosphate Inhibitor Cocktail 100x; EDTA 100x; all from Thermo Fisher Scientific, Waltham, MA, United States). The protein concentration in the lysates was determined using the BCA test (Pierce BCA Protein Assay Kits, Thermo Fisher Scientific, Waltham, MA, United States). Protein precipitation with 10% TCA acid (Sigma Aldrich, Poznan, Poland) at a ratio of 1:1 was performed via incubation on ice for 15 min. After that, the pellets were dried and suspended in 1.5x sample buffer (NZYtech, Lisboa, Portugal). Then, 25 µg of protein for each sample was applied to a 12% polyacrylamide gel, and SDS‒PAGE was carried out. After electrophoresis, the proteins from the gel were transferred to a 0.2 µm PVDF transfer membrane (Thermo Fisher Scientific, Waltham, MA, United States). The membrane was subsequently blocked with 5% nonfat milk (SM Gostyn, Poland) for 1 h, after which it was incubated with antibodies (Supplementary Table S1 **)**. Immunoimaging was performed via an enhanced chemiluminescence (ECL) kit (Bio-Rad, Hercules, CA, United States). The results obtained for all the samples used were normalized to the level of GAPDH.

### 2.7 RT‒qPCR analysis

The cells were stored at −20 °C in stayRNA^TM^ (A&A Biotechnology, Gdansk, Poland). Before the isolation of RNA, the cells were thawed, washed once with ice-cold PBS and then suspended in fenozol. Total RNA was extracted using Total RNA Mini Plus according to the manufacturer’s protocol (A&A Biotechnology, Gdansk, Poland). The samples were shaken 5 times (2 min, 0 °C, 1,500 rpm). The temperature was controlled between stages, and the samples were mixed by inversion. The concentration and purity of the RNA were assessed spectrophotometrically (A260/A280 method) using a NanoDrop One Spectrophotometer (Thermo Scientific, Waltham, MA, United States). Reverse transcription of 1 μg of total RNA was performed via the Reverse Transcription Reagent iScript^TM^ Advanced cDNA Synthesis Kit according to the manufacturer’s instructions (Bio-Rad, CA, United States). Real-time qPCR was performed with SsoAdvanced^TM^ SYBR® Green Supermix using CFX96 Connected^TM^ Real-Time PCR Detection System (both reagents and equipment–Bio-Rad, CA, United States). The RT‒PCR was run under the following cycling conditions: 2 min at 95 °C followed by 40 cycles of 5 s at 95 °C and 30 s at 60 °C. Each sample was analyzed at least in triplicate, and the results obtained after 32 cycles were considered events that were too rare to be classified (in this case events at Ct 40 were taken for calculations of statistical significance). Relative expression levels were determined via the comparative Ct method (ΔΔCT method), with *RPS18* as an endogenous control and the results for C3A cells as a calibrator. The specific primers purchased from Bio-Rad are listed in Supplementary Table S2.

### 2.8 Statistics

Statistical analysis was performed via Statistica 10 software (StatSoft Inc., Tulsa, OK, United States). Statistical significance was determined via one-way analysis of variance (ANOVA) and the Duncan *post hoc* test. The data are presented as the means ± SDs.

## 3 Results

### 3.1 Comparison of cell viability and doubling time

**FIGURE 1:**
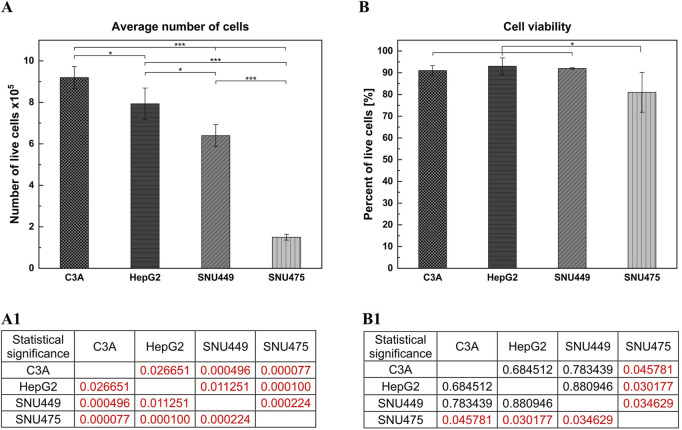
Number of live cells and cell viability of different liver tumor cell lines. Cells were seeded at a density of 1.5 × 10^5^ cells per well. The cell culture was performed on the 12-wells culture plate for 4 days without media change. The number of biological replications performed was 3. On the last day of the culture, cells were detached from the culture surface and counted with trypan blue. **(A)** Average number of cells. (A1) Statistical significance (p) for **(A)**. **(B)** Cell viability. (B1) Statistical significance (p) for **(B)**. The results were analyzed using the one-way analysis of variance (ANOVA) and Duncan *post hoc* test. Results are presented as mean value ±SD, n = 3. Level of significance: *p < 0.05, **p < 0.01, ***p < 0.001.

**FIGURE 2:**
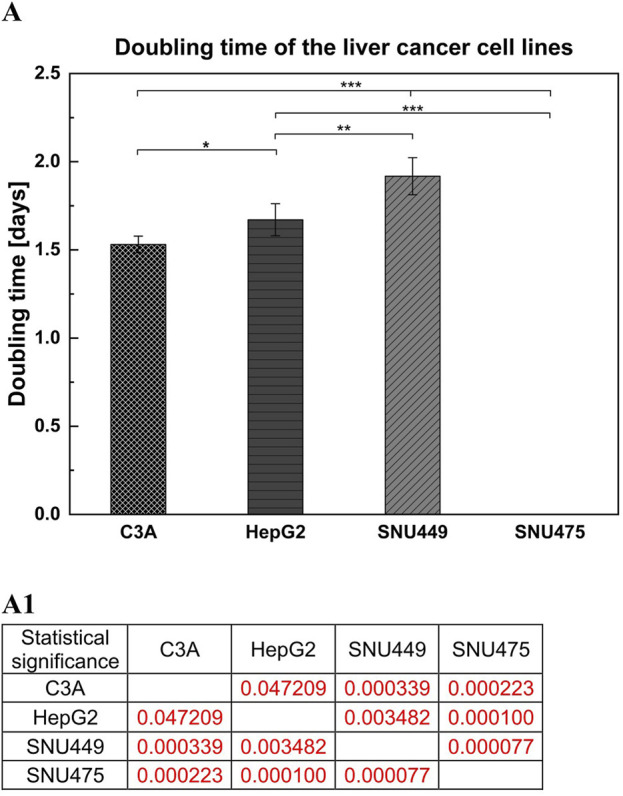
Doubling time of liver tumor cell lines. **(A)** For counting cells doubling time 1.5 × 10^5^ cells per well were seeded on 12-well culture plate. Culture was performed for 4 days without media change. (A1) Statistical significance (p) for **(A)**. The results were analyzed using the one-way analysis of variance (ANOVA) and Duncan *post hoc* test. Results are presented as mean value ±SD, n = 3. Level of significance: *p < 0.05, **p < 0.01, ***p < 0.001.

**FIGURE 3:**
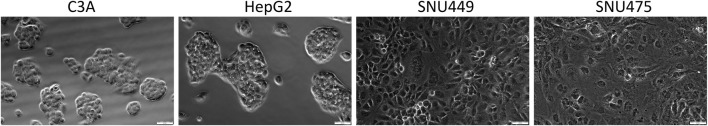
Morphology of liver tumor cells. Cells were seeded on 24-wells culture plate at the density of 8 × 10^4^ cells per well and cultured for 3 days. Microphotographs were taken using fluorescent microscope Olympus IX71, in visible light with magnification 20x.

After 4 days of culture without a medium change, the cells were detached by trypsinization and counted. The average number of cells (Figure 1A) showed that after 4 days of culture, the highest numbers were obtained for C3A, and the lowest were obtained for the SNU475 cell line. The cell viability was similar for C3A, HepG2, and SNU449 and was 90%, but for SNU475, it was 80% (Figure 1B). The cell doubling time was calculated as described earlier (Korzyńska and Zychowicz, 2008). The results revealed that the fastest dividing cells were C3A; for doubling, they needed 1.5 days, whereas SNU475, during 4 days of culture, did not divide at all (Figure 2A). A comparison of cell morphology revealed that C3A and HepG2 cells were smaller than both types of SNU and that the cells grew on top of each other, forming cell aggregates. SNU cells, especially SNU475 cells, were larger and more flattened and grew on the culture surface as a monolayer (Figure 3).

### 3.2 Cellular metabolic activity

**FIGURE 4:**
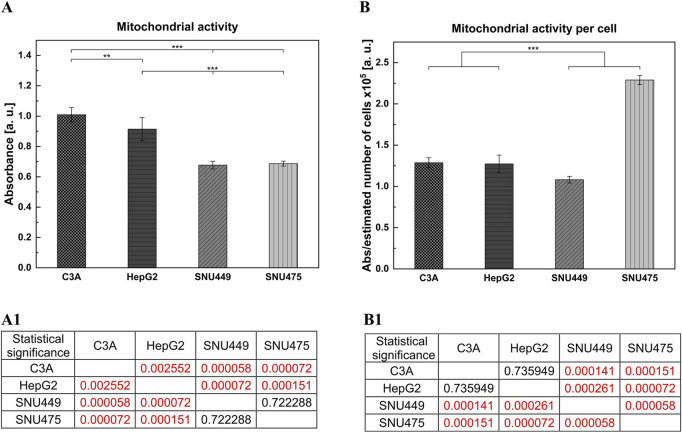
Cellular metabolic activity of liver tumor cells. Cells were cultured in dedicated to each cell line medium for 4 days without media change. Cells were seeded at the density of 3 × 10^4^ cells per well, on the 96-wells plate, and six biological replications were performed. Metabolic activity was performed with MTT test. **(A)** Cellular metabolic activity of liver tumor cells. The results were obtained for absorbance measurements. (A1) Statistical significance (p) for **(A)**. **(B)** Metabolic activity calculated for the estimated number of liver tumor cells. The obtained results showed metabolic activity per cell, absorbance obtained from MTT test was divided by estimated number of cells. (B1) Statistical significance (p) for **(B)**. The results were analyzed using the one-way analysis of variance (ANOVA) and Duncan *post hoc* test. Results are presented as mean value ±SD, n = 6. Level of significance: *p < 0.05, **p < 0.01, ***p < 0.001.

Analysis of cell metabolic activity was performed via the MTT test. The cells were cultured for 4 days without changing the medium. On the basis of the results obtained from only the absorbance measurements, we observed greater metabolic activity for C3A and HepG2 cells than for both SNU cell lines (Figure 4A). When the absorbance per number of cells was calculated, the highest metabolic activity was observed for SNU475 – the cells that did not divide during the time of the experiment (Figure 4B).

### 3.3 Albumin production

**FIGURE 5:**
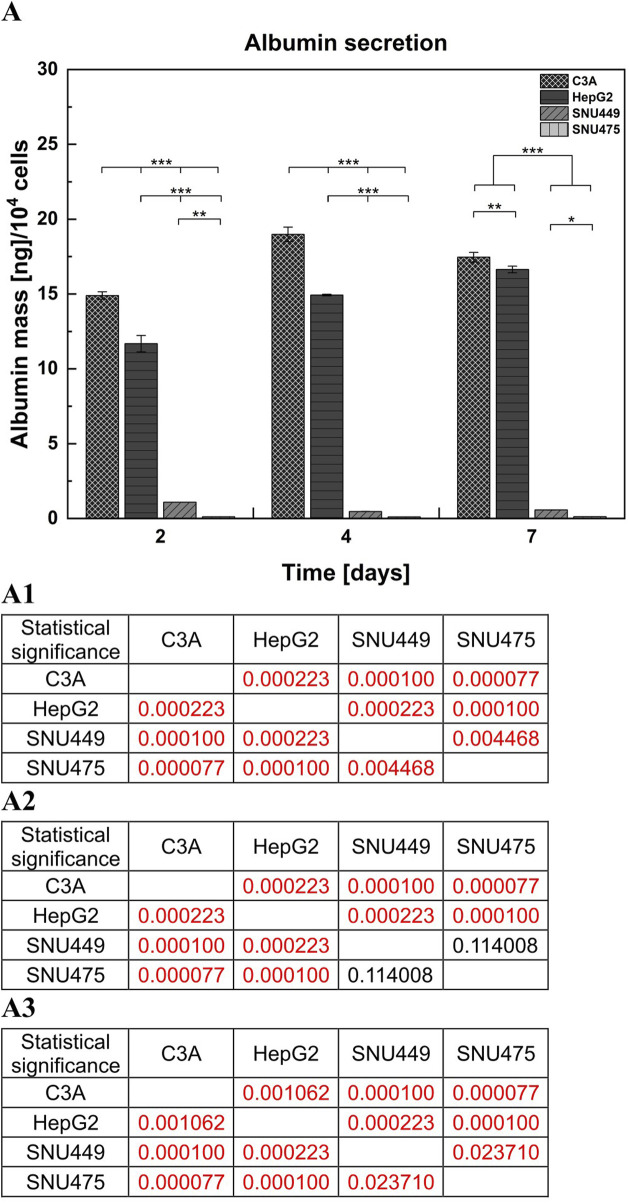
Albumin secretion by liver tumor cells. **(A)** The measurements of albumin production were performed with the use of sandwich ELISA test. Cells were cultured for 7 days on T25 culture flasks, 1 × 10^6^ cells were seeded on the culture surface. Medium samples were collected after 2, 4 and 7 days of culture. (A1) Statistical significance (p) for second day of culture for **(A)**. (A2) Statistical significance (p) for fourth day of culture for **(A)**. (A3) Statistical significance (p) for seventh day of culture for **(A)**. The results were analyzed using the one-way analysis of variance (ANOVA) and Duncan *post hoc* test. Results are presented as mean value ±SD, n = 3. Level of significance: *p < 0.05, **p < 0.01, ***p < 0.001.

Albumin is one of the most important and abundant proteins produced by hepatocytes. To detect and evaluate differences in albumin secretion between the tested cell lines, a sandwich ELISA was used. Albumin production in SNU449 was very low and decreased during culture. SNU475 did not produce albumin at detectable levels in contrast to the other cell types. Moreover, C3A during the 4 days of culture produced more albumin than did HepG2 (p < 0.001). However, on the seventh day of culture, both cell lines achieved similar levels of albumin production, but the difference was still statistically significant (p < 0.01) (Figure 5).

### 3.4 Formation of apical vacuoles

**FIGURE 6:**
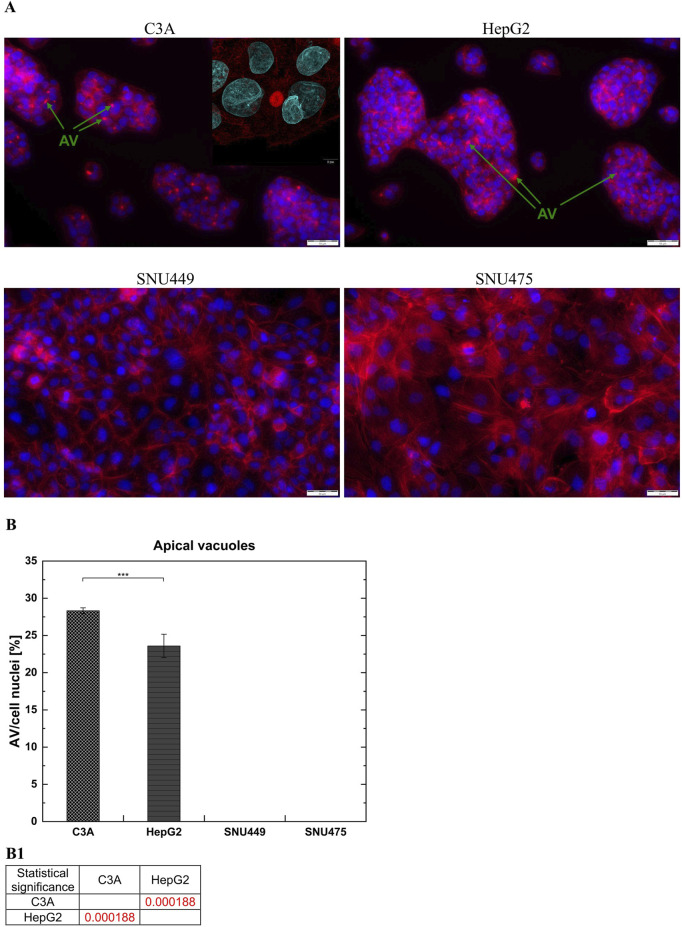
Apical Vacuoles (AV) in liver tumor cell cultures. **(A)** Images showed merged microphotographs of cell actin filaments stained with phalloidin (red) and nuclei with DAPI (blue). Cells were cultured for 3 days and then microphotographs were taken using fluorescent microscope Olympus IX71 at a magnification of 20x (scale bar 50 µm). Insert microphotograph was taken using confocal laser scanning microscope Fluoview FV4000 at a magnification of 60x (scale bar 5 µm). Apical vacuoles marked by green arrows have ring-like shape and are present between C3A and HepG2 cells. **(B)** Cell membrane polarization. The ratio of number of AV to number of cell nuclei in liver tumor cell lines was counted from five fields of view for every cell type at magnification 20x. (B1) Statistical significance (p) for seventh day of culture for **(B)**. The results were analyzed using the one-way analysis of variance (ANOVA) and Duncan *post hoc* test. Results are presented as mean value ±SD, n = 5. Level of significance: *p < 0.05, **p < 0.01, ***p < 0.001.

The presence of apical vacuoles (AV), which are formed between adjacent hepatic cells, indicates the degree of polarization of cell membranes and is therefore one of the parameters determining the degree of differentiation of these types of cells. AV are structural and functional precursors of the *bile canaliculi* in the liver. They display a ring shape and are present between the junctions of cell membranes. We used phalloidin staining to indicate the presence of AV in the cells and compared them with images from a fluorescence microscope. We observed the appearance of AV only between C3A and HepG2 cells (Figure 6A). Moreover, the results showed that, compared with HepG2 cells, the C3A cell line formed a greater number of AV (p < 0.001) (Figure 6B).

### 3.5 Protein expression

Using western blot, we analyzed synthesis of the following gene products: albumin (ALB), arginase 1 (ARG1), carbamoyl-phosphate synthase 1 (CPS1), alpha-fetoprotein (AFP), human nuclear factor 4 alpha (HNF4α) and human nuclear factor 1 alpha (HNF1α), as well as cytochrome P450 isoenzymes CYP3A4, CYP2E1, and CYP2C8. As a control, a glyceraldehyde 3-phosphate dehydrogenase (GAPDH) housekeeping gene product was used.

**FIGURE 7:**
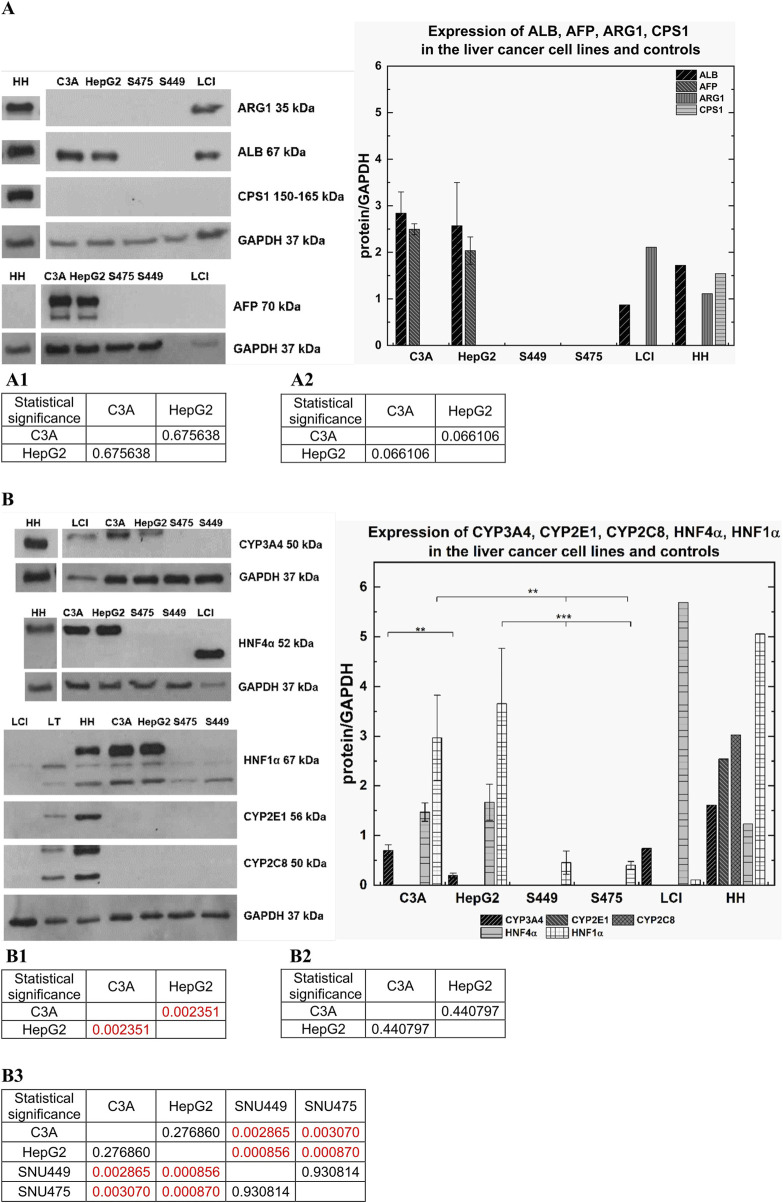
Comparison of protein expression in liver tumor cells. Western blot analysis was performed for C3A, HepG2, SNU475 (S475), SNU449 (S449) cell lines, Liver cell isolate (LCI), and human hepatocytes (HH). Per line was used 25 µg of protein. Samples were incubated with various antibodies. As a control for all performed WB analysis GAPDH protein was used–band size at 37 kDa. **(A)** Following gene products: ALB, AFP, ARG1, CPS1, GAPDH were analyzed for C3A, HepG2, SNU449, SNU475, LCI, and HH. **(A)** Left panel: Human Albumin (ALB) – predicted band size at 67 kDa. Arginase 1 (ARG1) – predicted band size at 35 kDa, Carbamoyl-Phosphate Synthase 1 (CPS1) – expected band size at 150–160 kDa, Alpha Fetoprotein (AFP) – predicted band size at 70 kDa. **(A)** Right panel: Graph showed expression of ARG1, ALB, CPS1, AFP for tumor cell lines and controls. (A1) Statistical significance (p) for ALB for **(A)** right panel. (A2) Statistical significance (p) for AFP for **(A)** right panel. **(B)** Products of following genes were analyzed: *CYP3A4, CYP2E1, CYP2C8, HNF4α, HNF1α, GAPDH*. **(B)** Left panel: Cytochrome P450 3A4 (CYP3A4) – predicted band size at 50 kDa. Cytochrome P450 2E1 (CYP2E1) – predicted band size at 52 kDa. Cytochrome P450 2C8 (CYP2C8) – predicted band size at 50 kDa. Hepatocyte Nuclear Factor 4 Alpha (HNF4α) – predicted band size at 52 kDa. Hepatocyte Nuclear Factor 1 Alpha (HNF1α) – band sizes at 80 kDa, 67 kDa, 61 kDa. **(B)** Right panel: Graph showed expression of CYP3A4, CYP2E1, CYP2C8, HNF4α, HNF1α for tumor cell lines and controls. (B1) Statistical significance (p) for CYP3A4 for **(B)** right panel. (B2) Statistical significance (p) for HNF4α for **(B)** right panel. (B3) Statistical significance (p) for HNF1α for **(B)** right panel. The results were analyzed using the one-way analysis of variance (ANOVA) and Duncan *post hoc* test. Results are presented as mean value ±SD, n = 3 for HCC cell lines, for LCI and HH n = 1. Level of significance: *p < 0.05, **p < 0.01, ***p < 0.001.

In the SNU449 and SNU475 cell lines, we did not observe bands specific for albumin. However, we observed a strong band of 67 kDa in size, which confirms the presence of albumin in C3A cells, HepG2 cells, human hepatocytes (HH), and Liver cell isolate (LCI). Moreover, C3A cells expressed higher levels of albumin than HepG2 cells did (not statistically significant) (Figure 7A), as shown by the results of a quantitative ELISA (Figure 5). At 35 kDa, a specific band for ARG1 was observed for HH and LCI. In turn, the CPS1 band at a size of 150 kDa was observed only for HH (Figure 7A). High expression of AFP was observed only in C3A and HepG2 cells. For both cell lines, the signal was observed as two bands at 70 kDa and 50 kDa, and C3A expressed slightly higher levels of this protein (not statistically significant) (Figure 7A). Bands for HNF4α at a size of 52 kDa were observed for HH, C3A, and HepG2 (differences not statistically significant), but a lower band at a size of 37 kDa was observed for the LCI. The HNF4α signal in the SNU cell lines was undetectable. LCI resulted in 3 times greater levels of HNF4α than HH, C3A, or HepG2 (Figure 7B). The highest CYP3A4 protein expression levels were observed in HH (band at 50 kDa). Twofold lower expression was observed for the LCI and C3A; for both, the signal levels were similar. For HepG2 cells, we observed a 3.5-fold lower signal than for C3A cells (p < 0.01). The SNU cell lines did not express CYP3A4 (Figure 7B). For HNF1α, we observed three bands: 80 kDa, 67 kDa, and 61 kDa for HH, C3A, and HepG2 cells, respectively. The signal for HH was 1.4 times greater than that for HepG2. The signal for HepG2 cells was greater than that for C3A cells, however this difference was not statistically significant. For the LCI, we observed a very weak signal—46 times lower than that of the HH. The HNF1α signal, from the 61 kDa and 67 kDa bands, was very low for the SNU cell lines. By comparing the signal of C3A with SNU, we obtained statistically significant results (p < 0.01). Also, a comparison of the SNU and HepG2 cells revealed that the difference was statistically significant (p < 0.001) (Figure 7B). At a band size of 56 kDa, corresponding to CYP2E1, we did not observe a signal in any of the hepatic cell lines, in contrast to the HH (Figure 7B). In the case of CYP2C8, similar to the results obtained for CYP2E1, the band at a size of 50 kDa was detectable only for HH (Figure 7B).

### 3.6 Gene expression analysis

The expression of the *ALB, ARG1, CPS1, HNF4α, HNF1α, CYP3A4, CYP2E1, CYP2C8, GLUL*, and *PXR* genes was evaluated via quantitative real-time PCR (RT‒qPCR). Gene expression was tested in C3A, HepG2, SNU449, SNU475, and HH. The expression data was normalized with respect to reference gene, *RPS18*, which appeared to be the most stable for cell lines among housekeeping genes tested in preliminary experiments (*RPS18, ACTB, GAPDH*).

**FIGURE 8:**
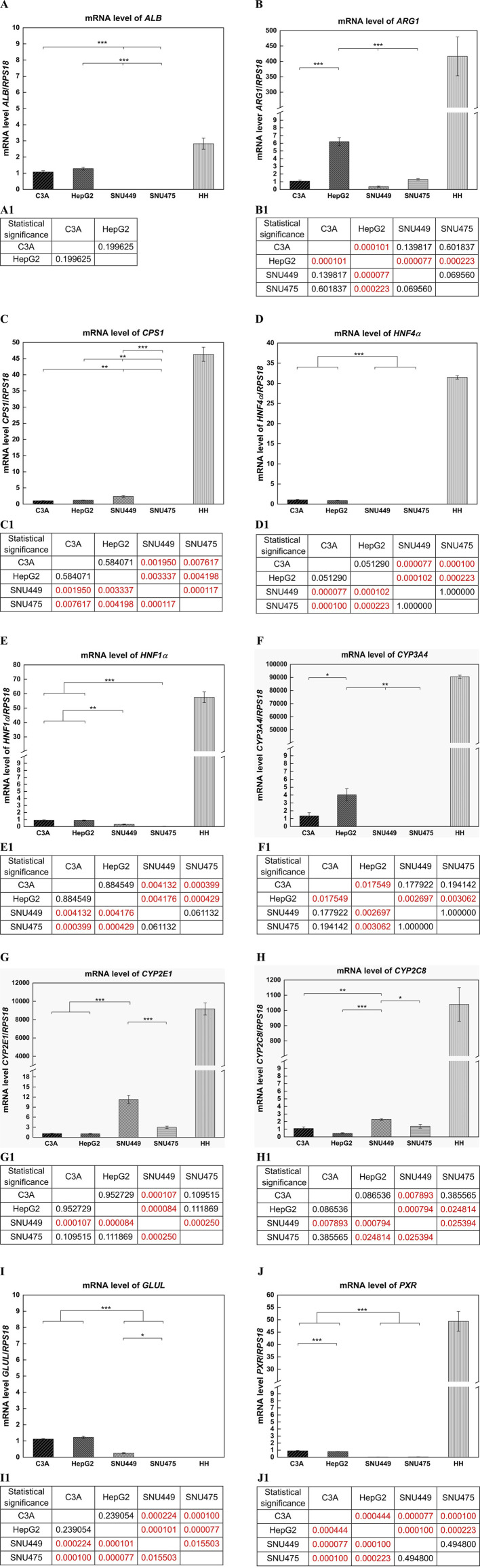
Gene expression analysis with use of quantitative real-time PCR (RT-qPCR). Results for *ALB*, *ARG1*, *CPS1*, *HNF4α*, *HNF1α*, *CYP3A4*, *CYP2E1*, *CYP2C8*, *GLUL*, *PXR* genes in liver tumor cell lines, and human hepatocytes (HH). Relative expression between samples was determined using the comparative Ct method (ΔΔCT Method), as a calibrator was used C3A cell line. The expression level of each gene was calculated as ratio of RQ obtained for each gene in type of cells to control - *RPS18*. For cancer cell lines each sample was analyzed in triplicate. **(A)** Results obtained for *ALB* gene. (A1) Statistical significance (p) estimated for cell lines for *ALB* for **(A)**. **(B)** Results obtained for *ARG1* gene. (B1) Statistical significance (p) estimated for cell lines for *ARG1* for **(B)**. **(C)** Results obtained for *CPS1* gene. (C1) Statistical significance (p) estimated for cell lines for *CPS1* for **(C)**. **(D)** Results obtained for *HNF4α* gene. (D1) Statistical significance (p) estimated for cell lines for *HNF4α* for **(D)**. **(E)** Results obtained for *HNF1α* gene. (E1) Statistical significance (p) estimated for cell lines for *HNF1α* for **(E)**. **(F)** Results obtained for *CYP3A4* gene. (F1) Statistical significance (p) estimated for cell lines for *CYP3A4* for **(F)**. **(G)** Results obtained for *CYP2E1* gene. (G1) Statistical significance (p) estimated for cell lines for *CYP2E1* for **(G)**. **(H)** Results obtained for *CYP2C8* gene. (H1) Statistical significance (p) estimated for cell lines for *CYP2C8* for **(H)**. **(I)** Results obtained for *GLUL* gene. (I1) Statistical significance (p) estimated for cell lines for *GLUL* for **(I)**. **(J)** Results obtained for *PXR* gene. (J1) Statistical significance (p) estimated for cell lines for *PXR* for **(J)**. Results were analyzed using the one-way analysis of variance (ANOVA) and Duncan *post hoc* test. Results are presented as mean value ±SD, n = 3 for liver tumor cell lines. Level of significance: *p < 0.05, **p < 0.01, ***p < 0.001. For HH n = 1, and statistical significance analysis was not performed.

Among cell lines, the highest level of the albumin gene expression was detected in HepG2 cells, which was 1.2 times greater than that in C3A cells (not statistically significant). For both SNU cell lines, albumin expression was not observed (p < 0.001) (Figure 8A). In HH, the level of *ALB* expression was 2.6 times greater than that of C3A (Figure 8A). The level of *ARG1* in HepG2 cells was high compared with that in other cell lines (p < 0.001), almost 5 times lower for SNU475 cells, 6 times lower for C3A cells, and almost 16 times lower for SNU449 cells (Figure 8B). For HH the level of expression of that gene was ca. 380 times greater than that of the C3A cells (Figure 8B). SNU449 presented the highest *CPS1* gene expression among cell lines, and a 2-fold lower level was observed for HepG2 and C3A cells (p < 0.01). For the SNU475 cells, gene expression was not detected (p < 0.001) (Figure 8C). The estimation of the level of *CPS1* expression revealed a 45-fold higher gene level for HH than for C3A (Figure 8C). Similar *HNF4α* gene expression results were obtained for C3A and HepG2 cells (difference not statistically significant). We did not observe gene expression in both SNU cell lines (p < 0.001) (Figure 8D). HH presented a 30-fold greater level of gene expression than C3A did (Figure 8D). In the case of *HNF1α*, the highest (similar) level of gene expression was obtained for C3A and HepG2 cells (difference not statistically significant) and was 3 times lower for SNU449 cells (p < 0.001). In SNU475, the expression of the analyzed gene was not observed (p < 0.001) (Figure 8E). HH presented a 64-fold greater level of the *HNF1α* gene expression than C3A cells did (Figure 8E). In cell lines, the highest level of *CYP3A4* gene expression was obtained for HepG2, which was 3 times greater than that of C3A (p < 0.05). For the SNU cell lines, the gene was not expressed (p < 0.01) (Figure 8F). HH presented very high levels of *CYP3A4* expression, more than 68,000 times higher than C3A did (Figure 8F). Another tested gene was *CYP2E1*, and we obtained the highest gene expression for SNU449, which was almost 4 times greater than that for SNU475 (p < 0.001)and nearly 11 times greater than that for C3A and HepG2 cells (p < 0.001) (Figure 8G). However, a ca. 8.300-fold greater level of expression of *CYP2E1* was obtained for HH than for C3A (Figure 8G). The last analyzed *P450* gene isoform was *CYP2C8*. The highest level of gene expression in cell lines was obtained for SNU449, which was 1.6 times lower for SNU475 (p < 0.05), 2 times lower for C3A (p < 0.01), and almost 5 times lower for HepG2 (p < 0.001) (Figure 8H). In turn, the level of *CYP2C8* expression was 945 times greater for HH than for C3A (Figure 8H). The highest expression of the *GLUL* gene was obtained for HepG2, whereas that of C3A was slightly lower (not statistically significant), and nearly 5 times lower levels were obtained for SNU449 (p < 0.001). The expression of *GLUL* was not observed neither in SNU475 (p < 0.001) nor in HH (Figure 8I). For the *PXR* gene, we obtained the highest (similar) expression levels in cell lines for C3A and HepG2 cells (p < 0.001), almost 33 times lower for SNU475 (p < 0.001), and 30 times lower for SNU449 (p < 0.001) (Figure 8J). The expression of that gene for HH was nearly 55 times greater than that for C3A (Figure 8J).

## 4 Discussion

In this study we compared several hepatoblastoma (C3A, HepG2) and HCC (SNU449, SNU475) cell lines, in terms of their behavior during culture and the differences between their phenotypes, including morphology and *in vitro* growth rate, as well as liver-related gene expression at the protein and mRNA levels. The comparison revealed noticeable differences between cell lines either within cells of the same cancer origin or between different types of cancer (hepatoblastoma or HCC). Notably, these cell lines, which are derived from various liver tumors, exhibit varying levels of invasiveness and distinct responses to anticancer treatments. Moreover, all tested cancer cell lines differed significantly from isolated human hepatocytes, which generally presented much greater gene expression levels.

Among all the tested cell lines, C3A had the fastest growth, with the shortest doubling time. In contrast, after 4 days in culture, there were ca. 6 times fewer SNU475 cells, suggesting that no doubling occurred during this time. Moreover, compared with other cell lines, SNU475 cells presented only slightly lower cell viability (80% vs 90% of living cells). In general, hepatoblastomas grow faster in culture than do HCCs (Figures 1, 2) and this is what determines their greater availability. Additionally, hepatoblastoma cultures presented greater cell metabolic activity than HCC cultures did, as measured via the MTT test, with the exception of SNU475 in the case when the absorbance was calculated per number of living cells. This finding might indicate that this cell line, despite its very low proliferation rate, is the most metabolically active among the tested cells (Figures 4A,B). Another physiological difference observed between these two cancer types is the tendency of hepatoblastomas to spontaneously form aggregates (so-called islands) whereas HCCs exhibit a more flattened phenotype (stronger adherence) (Figure 3). It is worth noting, that in preparation for hepato- or cytotoxicity assays, spheroids or organoids, which recapitulate the 3D architecture, heterogeneity, and cell functions of primary tissues, may be preferable forms of cell culture.

One of the most important hepatocyte functions, enabling the estimation of liver-specific cell functionality, is albumin production (Zakrzewska et al., 2017). In this context, C3A cells performed best and hepatoblastomas outperformed HCC cell lines. As shown by the results of the quantitative ELISA, SNU449 secreted minimal amounts of albumin into the culture medium, whereas in the SNU475 cultured medium, albumin production was not detected (Figure 5). Interestingly, in hepatoblastomas albumin synthesis increased during 7 days of culture, but in SNU449, it decreased. When the ability of the examined cells to form AV was tested, it appeared that hepatoblastomas formed these structures efficiently, yet the HCC cell lines were incapable of doing so (Figures 6A,B). This observation is important because the polarization of hepatocyte membranes enables intracellular transport, which is necessary for xenobiotic processing and detoxication. Notably, that according to our unpublished observations, isolated human hepatocytes dedifferentiate so quickly *ex vivo* that they are also unable to form bile canaliculi precursors.

Using western blot, a semiquantitative method for estimation of protein production, we checked the absence or presence, in the tested cell lines and hepatocytes, of the following proteins: albumin (ALB), which is produced exclusively by hepatocytes; arginase 1 (ARG1), one of the key proteins of the urea cycle; and carbamoyl-phosphate synthase 1 (CPS1), the major mitochondrial urea cycle enzyme. Moreover, we analyzed the expression levels of alpha-fetoprotein (AFP), which is specific for immature hepatocytes and the tumor cells; human nuclear factor 4 alpha (HNF4α) and human nuclear factor 1 alpha (HNF1α), which are transcription factors that regulate the expression of several liver-specific genes. We also verified the expression of the cytochrome P450 isoenzymes CYP3A4, CYP2E1, and CYP2C8, which are involved in the metabolism of xenobiotics (Figure 7). The profile of the expression of these proteins differs significantly between cancer types. The presence of albumin was confirmed only in HepG2 and C3A cells, as well as in human hepatocytes and liver cell isolate. As expected, AFP was not found in the HH or LCI but was also not present in the tested HCC cells and was found only in the analyzed hepatoblastomas. The production of urea cycle enzymes ARG1 and CPS1 was below detection levels in both cancers which is in accordance with the observations published by other authors (Missiaen et al., 2022). Interestingly, the protein band corresponding to ARG1 is visible in the HH and LCI preparations, whereas this band assigned to CPS1 is only visible in the HH preparations. The major liver regulatory proteins HNF4α and HNF1α were detected only in cultured HH and in both HepG2 and C3A hepatoblastoma cells. HNF4α, but not HNF1α, was also detected in LCI, but these proteins were not detected in SNU475 and SNU449 HCC cells. Similarly, the main liver cytochrome P450 isoenzyme, CYP3A4, was present in the HH, LCI, HepG2, and C3A preparations but not in the SNU preparations. However, the other two isoenzymes, CYP2E1 and CYP2C8, were found only in the HH.

At the mRNA level, the expression of the following liver-specific genes was tested via RT-qPCR: *ALB, ARG1, CPS1, HNF4α, HNF1α, CYP3A4, CYP2E1, CYP2C8, GLUL*, and *PXR*. The levels of transcripts were quantitatively measured in the C3A, HepG2, SNU449, and SNU475 cell lines and compared to the gene expression in isolated human hepatocytes (Figure 8). Generally, the results for *ALB* mirror the gene expression observed at the protein level. Compared with those of HH, the *ARG1, CPS1*, *HNF4α,* and *HNF1α* expression levels in tumor cell lines are minimal, if at all. The observations are also similar for all tested P450 isoenzymes and for *PXR*, the pregnane X receptor, which is a nuclear receptor of foreign toxic substances that upregulates the expression of proteins involved in the detoxification and clearance of these substances from the body. In summary, the RT-qPCR results revealed that the expression of the tested genes in tumor cells of both origins did not approach the levels observed in HH with the exceptions of *ALB* and *GLUL*. *GLUL* encodes glutamine synthetase, an enzyme involved in ammonia detoxification, but its function is not linked to the urea cycle. In this case, the transcript was detected at very low levels in C3A, HepG2, and SNU449 cells but not in SNU475 and HH. This finding is unexpected since its activity is usually observed in a subset of pericentral hepatocytes, and again, it might result from rapid HH dedifferentiation *in vitro*.

Despite their imperfections in recapitulating hepatocyte functionalities, the described cell lines, especially HepG2 and C3A, are widely used in basic research and drug testing. In contrast to HepG2 and its clonal derivative C3A, which is derived from hepatoblastoma (López-Terrada et al., 2009; Arzumanian et al., 2021), the SNU449 and SNU475 cell lines belong to the mesenchymal-like subgroups of HCC (Oz et al., 2021; Nguyen et al., 2022). Both SNU cell lines are commonly used as models of cancer cells to study the mechanisms of tumor development, biological activities, and pathways leading to cell death (Azbazdar et al., 2023; Suo et al., 2023; Baris and Demir, 2024). They are also commonly used to evaluate anticancer targets and the potential of experimental compounds (Lee et al., 2020; Sanaei et al., 2021; Abdou et al., 2023), drug nanocarriers (Xu et al., 2023; Pieretti et al., 2024), new nonpharmacological technologies (Saadawy et al., 2023), and well-known drug resistance mechanisms (Son et al., 2021).

The differences in response to the toxicity of anticancer drugs arise from the heterogeneity of individual cancer cell types. SNU475 cells are described in the literature as a model of a more aggressive cancer with higher migratory capability than less invasive HepG2, with SNU475 cells being more mesenchymal-like and HepG2 cells being more hepatoblast/epithelial-like (Caruso et al., 2019; Akbari et al., 2021; Nguyen et al., 2022). For example, in a study on how cytoskeletal dynamics regulate stromal invasion behavior in different liver cancer subtypes, the authors noted that cytoskeletal disruptions notably hinder highly migratory SNU475 cells, but have a less significant effect on more proliferative HepG2 cells (Nguyen et al., 2022). In a study by (Akbari et al., 2021) the authors showed that LGR5 expression—a marker important for liver regeneration and stem cell maintenance—was detected in hepatoblast-like lines such as HepG2, but was low or absent in mesenchymal-like lines, including SNU449 and SNU475. Activation of the canonical Wnt pathway significantly increased LGR5 expression in HepG2, but not in SNU449 or SNU475.

SNU449 and SNU475 cells exhibit different responses to chemotherapy. Consistent with the findings of (Mok et al., 2006), SNU449 demonstrated greater resistance to standard chemotherapeutic agents, such as paclitaxel, docetaxel, doxorubicin, and the most effective patupilone, than other HCC cell lines, including SNU475. Similarly (Sanaei et al., 2021), reported that SNU449 showed greater resistance to sodium butyrate than SNU475 (IC_50_: 6.976 µM vs 4.175 µM). Although apoptosis levels induced by both compounds were higher in SNU475, statistical differences between the cell lines were not assessed. In contrast, both lines exhibited similar sensitivity to 5′-fluoro-2′-deoxycytidine. In line with this, no significant differences were observed between SNU449 and SNU475 in response to plasma-activated air-driven water mist (Saadawy et al., 2023). On the other hand, in a study by (Son et al., 2021), HepG2 was the most sensitive to sorafenib, while SNU475 was the most resistant, likely due to differing expression levels of FGL1, a protein associated with sorafenib response. Interestingly, in response to ZnO nanoparticles, SNU449 cells were more sensitive than HepG2, as indicated by significantly lower IC_50_ values. Additionally, ZnO-induced cell death differed: apoptosis predominated in HepG2, while necroptosis occurred in SNU449 (Pieretti et al., 2024).

## 5 Conclusion

In summary, the response to chemotherapy differs among HepG2, C3A, SNU449, and SNU475 cells, which may be due, among other factors, to a different repertoire of active genes responsible for drug metabolism, including cytochromes, and to differences in cell physiology, such as growth behavior, albumin production and the ability to maintain membrane polarity (AV formation). In our study, we demonstrated, at both the protein and transcript levels, different levels of expression of the selected cytochrome P450 isoforms, namely, CYP2E1, CYP2C8, and CYP3A4, which are activated by different xenobiotics. Additionally, the *PXR* gene, which plays important roles in disposing of endogenous and xenobiotic compounds and maintaining metabolic homeostasis, was differentially expressed in the tested cell lines. Importantly, these levels were much greater in HH. Similarly, these differences were observed for hepatocyte nuclear factors (*HNF1α* and *HNF4α*), genes that regulate the synthesis of major liver-related proteins. Finally, they liver-relevant proteins, such as ALB (major liver-produced protein responsible for the binding of toxic compounds), ARG1 and CPS1 (enzymes involved in the detoxication of ammonium cations via the urea cycle), are present in different amounts in the tested cells. Considering the above factors, careful selection should be performed to choose the proper cell line to test particular xenobiotic-driven hepatotoxicity. Moreover, these results indicate that the SNU475 and SNU449 cell lines in particular might not be perfectly suited for drug testing. Nevertheless, HH constitute the best cell source for drug testing, and no alternative cell source can replace the functionality of these cells. On the other hand, HH are not widely available, quickly dedifferentiate *ex vivo* and do not guarantee reproducibility, because originate from patients with variable genetic backgrounds.

The main limitation of the presented study is the lack of functional testing of the investigated genes. Additionally, the inducibility of various cytochrome P450 isoforms was not analyzed. The study was also restricted to four cell lines, and the cultures were maintained in a monolayer. This experimental design was chosen to simplify the study as much as possible and to minimize the influence of multiple variables on the results. According to our assumptions, this approach allowed us to reduce interpretative errors resulting from the high genetic background variability—both among the studied cell lines and primary hepatocytes derived from different donors—and to present, in an intentionally simplified manner, conclusions regarding the variable suitability of cell lines of different origins for cytotoxicity studies.

## Data Availability

The original contributions presented in the study are included in the article/Supplementary Material, further inquiries can be directed to the corresponding author.
