# Supplementary material for: Variable expression of hepatic genes in different liver tumor cell lines: conclusions for drug testing
Source: Front Cell Dev Biol. 2025 Sep 2;13:1646602. doi: 10.3389/fcell.2025.1646602 (PMC12436404; doi:10.3389/fcell.2025.1646602)
Supplement: Supplementary file 1 [file DataSheet1.pdf]

Supplementary Material

1 Supplementary Tables

Supplementary Table 1. Table with the antibodies used.

**Antibody**	**Cat. number**	**Concentration**
**Primary antibodies**		
goat anti human Alb-HRP	Bethyl, A80-129P	1:10 000
mouse anti human HNF4A	Invitrogen, MA1-199	1:1000
mouse anti human HNF1A	Invitrogen, MA1-25417	1:500
rabbit anti human AFP	Invitrogen, MA5-16321	1:1000
rabbit anti human CPS1	Abcam, EPR7493-3	1:500
mouse anti human CYP3A4	Invitrogen, MA5-17064	1:1000
rabbit anti human CYP2C8	Proteintech, 16546-1-AP	1:5000
rabbit anti human CYP2E1	Invitrogen, PA5-35351	1:1000
rabbit anti human ARG1	Invitrogen, PA5-29645	1:5 000
rabbit anti human GAPDH	Invitrogen, PA1-988	1:10 000
**Secondary antibodies**		
goat anti mouse IgG Fc Cross-Adsorbed Secondary Antibody HRP	Invitrogen, 31439	1:2000
goat anti rabbit IgG H&L HRP	Abcam, ab97051	1:10 000

Supplementary Table 2. Specific primers used for RT–qPCR analysis (https://www.Bio-Radad.com/).

Gene Name	Gene Symbol	Gene Aliases	Unique Assay ID	Amplicon Length (bp)	Efficiency (%)
albumin	*ALB*	DKFZp779N1935, PRO0883, PRO0903, PRO1341	qHsaCID0017798	112	95
glutamate-ammonia ligase	*GLUL*	GLNS, GS, PIG43, PIG59	qHsaCED0001392	73	99
nuclear receptor subfamily 1, group I, member 2	*PXR*	BXR, ONR1, PAR, PAR1, PAR2, PARq, PRR, NR1I2, SAR, SXR	qHsaCED0045230	96	97
hepatocyte nuclear factor 1 homeobox A	*HNF1A*	HNF-1A, HNF1, IDDM20, LFB1, MODY3, TCF-1, TCF1	qHsaCED0001918	61	99
hepatocyte nuclear factor 4, alpha	*HNF4A*	FLJ39654, HNF4, HNF4a7, HNF4a8, HNF4a9, HNF4alpha, MODY, MODY1, NR2A1, NR2A21, TCF, TCF14	qHsaCID0015879	108	102
arginase, liver	*ARG1*	Not Available	qHsaCED0003959	67	100
carbamoyl-phosphate synthase 1, mitochondrial	*CPS1*	CPSASE1	qHsaCID0037920	143	98
cytochrome P450, family 2, subfamily C, polypeptide 8	*CYP2C8*	CPC8, CYPIIC8, MP-12/MP-20	qHsaCID0021321	119	96

cytochrome P450, family 3, subfamily A, polypeptide 4	*CYP3A4*	CP33, CP34, CYP3A, CYP3A3, CYPIIIA3, CYPIIIA4, HLP, MGC126680, NF-25, P450C3, P450PCN1	qHsaCID0012316	98	101
cytochrome P450, family 2, subfamily E, polypeptide 1	*CYP2E1*	CPE1, CYP2E, P450-J, P450C2E	qHsaCED0044326	72	93
ribosomal protein S18	*RPS18*	D6S218E, HKE3, KE-3, KE3, MGC117351, MGC126835, MGC126837, S18	qHsaCED0037454	67	94
